# Effects of the Generic Masculine and Its Alternatives in Germanophone Countries: A Multi-Lab Replication and Extension of Stahlberg, Sczesny, and Braun (2001)

**DOI:** 10.5334/irsp.522

**Published:** 2024-10-01

**Authors:** Hilmar Brohmer, Gabriela Hofer, Sebastian A. Bauch, Julia Beitner, Jana B. Berkessel, Katja Corcoran, David Garcia, Freya M. Gruber, Fiorina Giuliani, Emanuel Jauk, Georg Krammer, Smirna Malkoc, Hannah Metzler, Hanna M. Mües, Kathleen Otto, Rima-Maria Rahal, Mona Salwender, Sabine Sczesny, Dagmar Stahlberg, Wilken Wehrt, Ursula Athenstaedt

**Affiliations:** 1 Department of Psychology, University of Graz, Graz, AT; 2 Study Centre for Health Science & Management, Baden-Württemberg Cooperative State University, Stuttgart, DE; 3 Department of Psychology, Goethe University Frankfurt, Frankfurt am Main, DE; 4 Mannheim Centre for European Social Research, University of Mannheim, Mannheim, DE; 5 University of Konstanz, Konstanz, DE, Complex Science Hub Vienna, Vienna, AT; 6 Department of Psychology, University of Salzburg, Salzburg, AT; 7 Department of Psychology, University of Zurich, CH; 8 Institute of Clinical Psychology and Psychotherapy, Technical University of Dresden, Dresden, DE; 9 Department of Medical Psychology, Psychosomatics and Psychotherapy, Medial University of Graz, Graz, AT; 10 Institute of Business and Vocational Education, Johannes Kepler University Linz, Linz, AT; 11 Institute for Education Practice and Practitioner Research, University College of Teacher Education Styria, Graz, AT; 12 Linz School of Education, Johannes Kepler University Linz, Linz, AT; 13 Complex Science Hub, Vienna, AT; 14 Medical University of Vienna, Vienna, AT; 15 Institute of Globally Distributed Open Research and Education, AT; 16 Center for Public Health, Department of Social and Preventive Medicine, Medical University of Vienna, Vienna, AT; 17 Department of Clinical and Health Psychology, University of Vienna, Vienna, AT; 18 Department of Psychology, Philipps University of Marburg, Marburg, DE; 19 Max Planck Institute for Research on Collective Goods, Bonn, DE; 20 School of Social Sciences, University of Mannheim, Mannheim, DE; 21 Institute of Psychology, University of Bern, Bern, CH; 22 Mannheim Center for European Social Research, University of Mannheim, Mannheim, DE; 23 Department of Work and Social Psychology, University of Maastricht, NL

**Keywords:** gender-inclusive language, gender-fair language, generic masculine, open data, multi-site study

## Abstract

In languages such as German, French, or Hindi, plural forms of job occupations and societal roles are often in a generic-masculine form instead of a gender-inclusive form. Although meant as ‘generic,’ this generic-masculine form excludes women from everyday language. Specifically, listeners and readers are less likely to think of women when this form is used. Due to the societal relevance of gender-inclusive language, we directly replicated and extended a classic study by Stahlberg, Sczesny, and Braun (2001, Experiment 2) in a multi-lab setting and as a registered confirmatory report. We prompted participants from German-speaking countries to name up to three celebrities each in six categories (e.g., ‘Name three politicians’ or ‘(…) singers’). We then counted how often participants mentioned women. Participants were either prompted with the generic-masculine form, a neutralized control form or one out of three gender-inclusive forms. Our data from twelve labs and *N* = 2,697 participants replicated the original effect: when prompted with gender-inclusive forms participants mentioned more women than when the generic masculine and the control form were used. Moreover, the effect remained present in multilevel models and when controlling for participants’ sex and their perceived base rate in these celebrity categories (i.e., the expected proportion of women). Other variables, such as political orientation or preference for gender-inclusive language, did not show large effects, either. We discuss the differences between specific gender-inclusive forms (e.g., the internal-I vs. feminine-masculine forms), implications for regulations and guidelines, as well as implications for non-binary and gender-diverse people.

## Introduction

The idea that language equates or influences the way we think and how we behave has a long history in social-cognitive psychology (von Humboldt 1843; Whorf 1956). The weak form of linguistic relativity—that is, that language affects the way we think—seems to have garnered some support. Several studies suggest that people’s perception of time (Boroditsky 2001), interpretation of events (Athanasopoulos et al. 2015), color perception (Winawer et al. 2007), or in-group and out-group biases (Danziger & Ward 2010) may vary according to how these concepts are verbally described. However, many of these earlier studies are either underpowered or were conducted across (rather than within) cultures, introducing many confounding variables. One of the few investigations to study linguistic relativity within languages failed to demonstrate any effect (IJzerman et al. 2015).

Language also seems to affect how people think about gender in the social world: In many languages, it is common to apply masculine words to refer to people of all genders—the so-called generic masculine. It is possible that the generic masculine may lead people to think less frequently about women or people who identify themselves neither as male nor as female in various contexts, such as when thinking about who is a typical doctor or scientist (for a review see Sczesny et al. 2016).

The generic masculine is potentially most explicit in gender-inflected languages like German, French, Hindi, Serbian, Zande (in Sub-Saharan Africa), or Spanish, in which nouns have specific grammatical genders (Stahlberg et al. 2007). Gender-inflected languages often include gender-specific versions of pronouns and nouns that describe certain societal roles or occupations (e.g., *the doctor* in German: ‘der Doktor’ is male and ‘die Doktorin’ is female; in French: ‘le docteur’ is male and ‘la doctoresse’ is female).

Using the generic masculine has been criticized by cognitive scientists and psychologists for several decades as they argue that it entrenches gender-stereotypical ideas about roles and occupations in society (e.g., Braun et al. 2007; Gastil 1990; Moulton et al. 1978; Stokes 2020). Specifically, people may think less frequently of women being in specific professions, when, for instance, in German the plural form ‘die Doktoren’ (*the doctors* in the generic masculine form) is used instead of ‘die Doktorinnen und Doktoren’ (*the female and male doctors*, the feminine-masculine word-pair form). Several experimental studies have supported this notion two to three decades ago (e.g., Braun et al. 1998; Gastil 1990; Stahlberg et al. 2001), although the evidential value of these studies is still unclear to date. With the advent of the replication crisis, doubts may emerge as to the stability and robustness of these effects, particularly as some studies in the literature were underpowered. Despite the societal relevance of this research, none of these conceptually similar, but methodologically different studies have been robustly and closely replicated to our knowledge. This is what we aim to change with this Confirmatory Report.

### Evidence of the Generic-Masculine Effect

A considerable body of research indicates that the generic masculine is not always read generically, that is, that it does not seem to make people think of both men and women in equal proportions. Early on, Moulton and colleagues (1978) and Gastil (1990) demonstrated for the English language that the generic usage of male pronouns like ‘he’ or ‘his’ is largely associated with mental representations of men. This work was extended and complemented by similar findings on gender-specific nouns in gender-inflected languages (for a review see Sczesny et al. 2016). As an example, Gygax et al. (2008) conducted a study in German and French and suggested that when a group was referred to in the generic masculine, participants thought it was more likely that the group consisted of men than women. This effect seemed to be independent of whether the group had a stereotypical male or female profession. In a similar vein, Rothmund and Scheele (2004) showed that German texts written in the generic masculine evoke more male than female representations.

Much of the work on the impact of the generic masculine in the German language has been conducted by the group of Stahlberg and colleagues. With different paradigms (e.g., Braun et al. 1998; Stahlberg & Sczesny 2001), the authors suggested that people thought more about men than women when exposed to nouns in the generic-masculine form, independent of their own sex (for similar findings but with a significant participant sex effect, see Gabriel & Mellenberger 2004).^1^ Consequently, associations related to alternative concepts (i.e., women) might be less likely to get activated or even inhibited. The authors then demonstrated that this generic-masculine effect could be considerably reduced through the use of gender-inclusive alternatives, when either both genders were explicitly referred to (feminine-masculine form) or when the so-called internal-I form^2^ was used to avoid long formulations (e.g., ‘die DoktorInnen’ or *the doctors*).

These findings could bear serious implications. In psychological research, the generic masculine might also have unintended effects relevant to research and assessment by affecting responses to self-report questionnaires (Vainapel et al. 2015). In a study on job ads, including only male pronouns—as compared to gender-inclusive language—induced a lower expected sense of belonging, less motivation to pursue the job, and lower identification with the job in US female undergraduates (Stout & Dasgupta 2011). Similar negative implications could already be present in primary school children as a study by Vervecken et al. (2013) suggests: When presented with job titles of stereotypically male occupations (e.g., pilots) in the generic-masculine (compared to the feminine-masculine form), children not only named female jobholders less frequently but also perceived women as less likely to succeed in these positions. Crucially, girls reported less interest in these jobs when the generic masculine was used. Another study suggested that male applicants were perceived as more suitable for high-status positions than female applicants when the generic masculine but not when the feminine-masculine form was used for the job title of a job advertisement (Horvath & Sczesny 2016, but see Castilla & Rho, 2023).

### Public Debate

Despite the evidence in favor of gender-inclusive language, its use in formal language has been debated for a long time. As the present replication effort is located in Germanophone countries, we are particularly aware of the debates in this area. In Germany, the Council for German Orthography declared that the use of the internal-I form (e.g., the application of the German plural form ‘DoktorInnen’ for both males and females) diverges from the orthographic norm but is not ‘wrong’ per se (Rat für deutsche Rechtschreibung 2016) and the largest German dictionary Duden recently released a guideline for gender-inclusive language use (Duden 2020). In order to make its use official in the future, the Swiss Federal Chancellery of Switzerland and the Austrian Ministry of Labor, Social Affairs and Consumer Protection released formal tutorials for the correct application of gender-inclusive language (BMASK 2015; Bundeskanzlei 2013). However, critiques have emphasized potential problems with imposed rules for language and literature expressing concerns about an incompatibility with grammar. For instance, it has been argued that the now common gender-star form (‘Doktor*innen’), which is meant to not only include men and women but also people who identify themselves as gender-non-binary,^3^ hinders readability (Düker 2018; Knoke 2017; Zeit Online 2021). This point is also often made for the other gender-inclusive language forms (see Rat für deutsche Rechtschreibung 2021; for the counter-argument that alternative forms do not inhibit readability see Friedrich & Heise 2019).

Sczesny and her colleagues (2016) have provided an overview of the evidence in favor of different language forms of gender-inclusive language and concluded that applying them indeed has the potential to reduce gender stereotyping and discrimination in society. However, they acknowledge that gender-inclusive language is seen negatively by some members of society, which is in accordance with the results of recent representative surveys in Germany (Infratest Dimap 2020, 2021). It is therefore an important open question whether there are similarly positive effects of gender-inclusive language for people who are more critical of gender-inclusive language or who are non-progressive in their political views.

### The Original Study and the Present Research

For this Confirmatory Report we conducted a large-scale replication of a seminal study that demonstrated the cognitive effect of the generic masculine and its alternatives (Stahlberg et al. 2001, Experiment 2). There were several reasons why it was deemed important to replicate such an experiment. First, a powerful replication of the original findings can underline the importance of adaptations in formal language, providing a more solid scientific ground for the acceptance (and less ideological resistance) of gender-inclusive language in society. Second, as many European societies have changed toward more liberal and gender-inclusive values throughout the last decades (Pinker 2018), it would be interesting to see if the original effect still holds today and to identify potentially relevant moderators (such as political orientation). Finally, the field of psychology calls for systematic replication studies as it undergoes a large crisis of credibility of previous findings (Open Science Collaboration 2015), and the findings on gender-inclusive language are no different. Several of the findings we reviewed have been underpowered (see Table S3, https://osf.io/76un5/) and the status of the evidential value of prior work is therefore unclear. Replication efforts are particularly urgent for politically and socially relevant effects so that they can inform future interventions. We believe that the effect of the generic masculine and its alternatives is precisely such an effect.

The original authors (Stahlberg et al. 2001, see also Braun et al. 2005) understand the effect of the generic masculine as a social-cognitive retrieval process: Using it for describing societal roles and categories in speech and writing should make the concept of ‘man’ and related associations more cognitively accessible in the recipient (i.e., the listener or reader), leading to a higher retrieval rate of male exemplars. By contrast, using gender-inclusive forms should lead to the cognitive retrieval of both men and women. Stahlberg and colleagues (2001, Experiment 2) tested this idea in a compelling study: They had participants list three celebrities in four categories (sports, politics, television, and music), with the gender-based forms of the instructions varying randomly across participants. Specifically, the authors contrasted the generic-masculine form with two alternatives (the internal-I form and the feminine-masculine form) as their most important effect. Participants came up with more women across categories when the alternative gender-inclusive forms were used compared to when the generic-masculine form was. With *d* = 0.59, 95%CI [0.14, 1.04],^4^ this is considered a medium effect according to both common thresholds (Cohen 1988) and empirically derived thresholds (Lovakov & Agadullina 2021), but it may be overestimated as indicated by its large confidence interval.

The study by Stahlberg and colleagues (2001) differs from comparable work in some important ways. It was the first study in the German language that investigated the effects of the generic masculine on memory retrieval. Participants named celebrities under the impression that the purpose of the study was to test their media knowledge, when in reality the number of women mentioned was the relevant outcome. In contrast, other seminal studies directly asked participants for the estimated percentage of women in certain categories (e.g., Braun et al. 1998). These indirect measures as used in Stahlberg et al. (2001) are not only less susceptible to consciously distorted answers but also closer to real-life situations in which the generic masculine could have an effect (e.g., naming people for a promotion). Moreover, other research focused only on specific job categories (e.g., politics, see Stahlberg & Sczesny 2001), making it impossible to determine whether the effects of the generic masculine generalize to other social categories and professions. Thus, the paradigm of Stahlberg et al. (2001) is particularly well-suited for a replication.

These assets notwithstanding, the study also came with some methodological limitations. Most prominent is its relatively small sample size of *N* = 90 for a between-participants design with three groups. While small samples were typical for that time—and some other studies in this area likely also suffered from low statistical power (see Table S3, https://osf.io/76un5/)—they cast some doubt on the robustness of the reported effects. Small samples are associated with less precise estimates of population effects (e.g., Kelley & Maxwell 2003) and generally tend to inflate the observed effects (e.g., Button et al. 2013). A large-scale replication enabled us to provide a more precise estimate of the effect of the generic masculine on the retrieval of women. Moreover, Stahlberg et al. (2001) only controlled for the effect of participants’ sex, but did not test for other potentially relevant control variables and moderators (such as participants’ political orientation)—another limitation that we addressed in the present endeavor.

With the present Confirmatory Report, we conducted a large-scale replication across twelve sites (see Table 1 and S1; https://osf.io/76un5/). In addition to the theoretical considerations speaking for a replication of this study, the paradigm is also straightforward, concise, and easy to implement online. Like in the original experiment, we measured the activation of concepts related to men and women via a listing task. Specifically, we counted the number of male or female exemplars participants mentioned when asked to list celebrities in certain societal categories (e.g., politics or sports). As an experimental intervention, the language form of those occupations varied between participants.

**Table 1:** Summary of the main hypotheses, models, variables, and effects of interest.


HYPOTHESIS	CONCEPTUAL MODEL	MODEL AND VARIABLES	EFFECT(S) OF INTEREST	REMARK

1. Compared to the **generic masculine** form, the **internall and feminine masculine** form will yield a higher number of women mentioned.	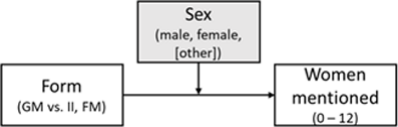	General linear model (ANOVA); IV: form, moderator: participant sex, DV: women mentioned^i^; additional multilevel model with Poisson-distributed measures per category nested in participants and labs	Helmert contrast (GM vs. II & FM); Cohen’s *d* for the mean difference	Close replication (Stahlberg et al., 2001, Experiment 2) Original categories: athletes, politicians, singers, tv hosts Three celebrities per category are required

2. Compared to the **generic masculine and the control** form, the **internal-I, feminine masculine**, and **gender star** form will yield a higher number of women mentioned.	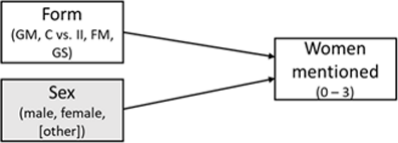	Multilevel model with Poisson-distributed measures per category nested in participants and labs, IV1: form, IV2: participant sex^ii^, DV: women mentioned	Deviation contrast (GM & C vs II. FM & GS); standardized effect: incident rate ratio	Based on Pre-Study 1 and 2 Original plus extra categories: writers and actors Two celebrities per category are required

3. Higher scores on the **perceived base rate** (perceived higher proportion of women) are associated with a higher number of women mentioned, when it is controlled for the form effect.	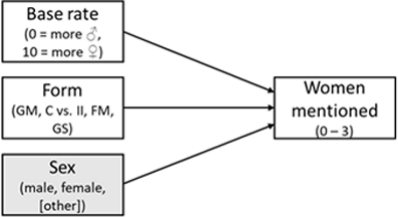	Multilevel model with Poisson-distributed measures per category (I1) nested in participants (I2), IV1: form, IV2: perceived base rate, DV: women mentioned	Effect of the perceived base rate (level 1) and of the form as in H2 (level 2); standardized effect: incident rate ratio	Based on Pre-Study 2 Original plus extra categories: writers and actors Two celebrities per category are required complete perceived base rate items


*Note*: GM = generic masculine, C = control, II = internal I, FM = feminine-masculine, GS = gender star; ^i^ an additional multilevel model will be calculated for Hypothesis 1, ^ii^ a language form × sex interaction will also be checked for Hypothesis 2, but effects will be taken from the covariate model; variables in gray boxes are controlled for, but not of primary interest; this table is revised and the original table can be found in Supplemental Materials 3 (https://osf.io/ecpgx).

**Figure 1:**
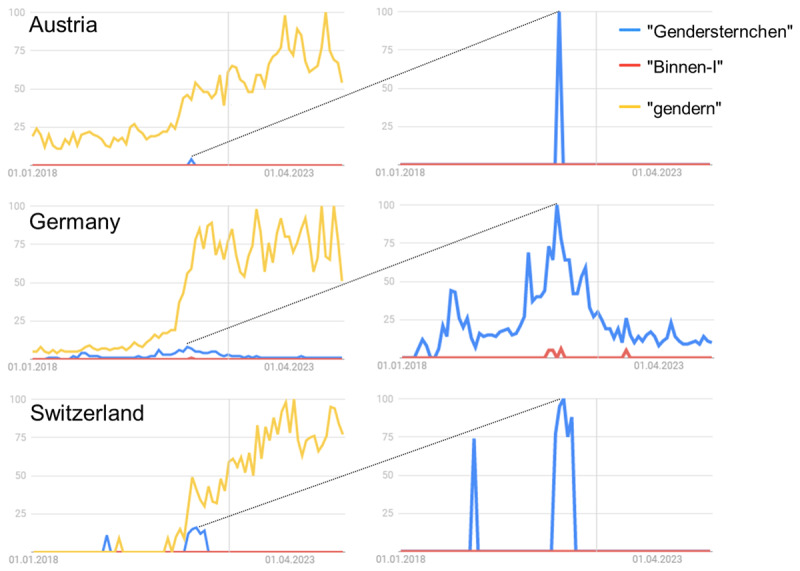
Google searches for ‘gendern’ (using gender-inclusive language in everyday language), ‘Binnen-I’ (internal I) and ‘Gendersternchen’ (gender star) from Jan 2018 to June 2024. *Note*: Based on a population of >60 million German users, >7 million Austrian users, and >8 million Swiss users; updated figure and original figure is in Supplemental Materials 3, https://osf.io/ecpgx; searches in percent are standardized on the maximum search per country; diagonal lines across panels connect reference maxima; absolute number of searches is not provided by Google Trends; example search for upper left panel: https://trends.google.de/trends/explore?date=2018-01-012024–05–18&geo=AT&q=Gendersternchen,Binnen-I,gendern.

We also extended the original study by adding another gender-inclusive condition: the gender-star form. While there seems to be a general increase of interest in gender-inclusive language (or ‘gendern’ in German) in German-speaking countries, the gender star has recently achieved more popularity than the internal-I (see Figure 1). Additionally, we addressed three aspects that might conceivably influence the listing of male and female exemplars and led us to the inclusion of additional variables.

The first aspect concerns the degree to which participants associate the respective occupations with either men or women, which relates to the availability heuristic (Tversky & Kahneman 1973) and related stereotypical ideas about gender roles (e.g., the expectations that men should be in high-status positions and the breadwinners in the household, while women should stay home and take care of the children; for psychological implications see Fiske et al. 2007; Schmitt 2015; Su et al. 2009; Wood & Eagly 2012). We assume that these views will be mirrored in a *perceived base rate* (Weber & Hilton 1990). This perceived base rate—the assumed proportion of men or women in specific roles—is an aspect that the original study did not examine. Indeed, in the original study, participants were asked to list three politicians, but they were not asked how many male and female politicians they usually encounter when they watch TV or read the news. We argue that this constitutes a crucial piece of information to control for. If one encounters more female politicians in general through the media, one might also be more likely to think of female politicians in a listing task.

The second aspect concerns potential cultural and societal changes since the original study was conducted. On the one hand, European societies have largely changed toward more liberal, progressive, and gender-inclusive values and rights in the last decades (Welzel 2013; Chapter 15 in Pinker 2018). On the other hand, societies have witnessed a backlash by right-wing movements in recent years, culminating in an increase in support for populist parties (e.g., Aisch et al. 2017; Rodrik 2020; Wodak & Krzyżanowski 2017). We argue that these developments and events could increase the variance of participants’ reactions to the task compared to 20 years ago. Especially, people who would identify themselves as politically left or who endorse equality and gender-inclusive language may respond in relatively unpredictable ways. Confronted with the generic-masculine form, they may either not identify this form as generic (by thinking that only men are meant) and hence only come up with male exemplars. But they may also feel reactant toward this form and deliberately only write down female exemplars or be indecisive about how to interpret this form. At the same time, participants from the right-wing political spectrum could also feel reactant toward the internal-I, feminine-masculine, or gender-star form, resulting in biased scores toward male exemplars. Building upon these considerations, we wanted to explore the effects of some possible moderator variables: *political orientation, attitudes toward gender-inclusive language, social-dominance orientation*, and *preference for socio-economic equality*.

The third aspect was already pointed out in the original study. It is possible that the effect between the generic masculine and the alternative forms is only driven by the generic masculine increasing the availability of male exemplars (thereby reducing the number of female exemplars) in the mind of the recipient (Braun et al. 2005). If this is true, then in a *control group*, in which no specific gender form will be presented (sometimes called neutralized form, see Sczesny et al. 2016; see also next section), women should be mentioned more often than in the generic-masculine group. In contrast, if the generic-masculine form and the neutralized form yield similar means, this implies that using gender-inclusive alternative forms helps activate the concept of women (which is otherwise not activated by default).

Before conducting the multi-lab study, we ran two pre-studies to address some of these potentially influential factors, improve our design, and refine our hypotheses. The full information on these pre-studies can be retrieved here: https://osf.io/kbynp/ (preregistrations: https://osf.io/5a7hw/ and https://osf.io/shknj/). The methods and materials used were similar to the ones we used in this multi-lab study.

### Summary of Pre-Study 1 & 2

Pre-Study 1 aimed to provide a first test of potential associations between the perceived base rate (How many men/women are active in the given profession?) and the number of men and women named in the listing task. We presented the listing task in a neutralized form (e.g., ‘Please list three persons in the domain of politics’) to obtain a first estimate for the control group. Moreover, we set out to test potential order effects in our main measure (i.e., the listing-task) and the perceived base rate, since asking participants how many women work in a specific profession may influence the number of women they come up with at the later listing task or vice-versa.

The findings indicated that asking participants about the perceived base rate first affected their responses in the following listing task. Therefore, we decided to present the listing task before the perceived base rate in the main study. Moreover, multilevel models of analysis revealed that there was a positive association between the number of women listed for a given profession and the perceived base rate of women for this occupation. This indicated that our perceived base rate measure might be an important predictor of the number of women named in our main task.

In Pre-Study 2, we conducted a first replication of Stahlberg et al. (2001) to obtain an estimate of the effects of different forms (i.e., generic masculine, internal I, feminine-masculine) on the number of women mentioned and to evaluate a number of potential moderators. Additionally, we modified the original paradigm by adding two more popular celebrity categories (writers and actors) to reduce the potentially detrimental effect of a lack of knowledge of women in a category on the number of women mentioned (as indicated by the significant effect of perceived base rate in Pre-Study 1). We tested whether the perceived base rate, or participants’ sex,^5^ political orientation, or attitudes toward gender-inclusive language were relevant covariates or moderators.

We replicated Stahlberg and colleagues’ (2001) findings in that people in the combined alternative groups (i.e., internal-I and feminine-masculine) listed more women than participants in the generic-masculine group, *d* = 0.78, 95%*CI* [0.65, 0.91]. Additionally, only the perceived base rate explained variance in the model next to the effect of gender form. This was surprising given the strong arguments for the potential relevance of the other variables. Since we could not eliminate the possibility that at least some of these results were due to the specifics of this pre-study or the examined sample, we nevertheless decided to re-evaluate the effects of participants’ sex, their political orientation, and their attitudes toward gender-inclusive language in the multi-lab study.

Finally, we compared the data from Pre-Study 1 and 2 and found that people who received the neutralized form (Pre-Study 1) listed a similar number of women as people who received the generic-masculine form (Pre-Study 2). This could indicate that the generic-masculine form might not necessarily induce people to think of more men, but rather that the alternative forms actively promote the retrieval of female exemplars.

### Hypotheses

Based on our theoretical considerations and the information obtained in the pre-studies, we defined three hypotheses for the multi-lab study. They are presented in Table 1 along with their conceptual models and effects of interest.

*Hypothesis 1* refers to a close replication of the original study (Stahlberg et al. 2001): We hypothesized that the number of listed female exemplars across categories (dependent variable: *number of women mentioned*) would be higher when participants read the gender-inclusive forms, that is, the feminine-masculine (e.g., ‘Politikerinnen und Politiker’ – *female and male politicians*) and the internal-I form (e.g., ‘PolitikerInnen’), compared to the generic-masculine (e.g., ‘Politiker’ – *politicians*) form. In line with the original study, this hypothesis was examined in a 2 (participant sex: male, female) × 3 (language form: generic masculine, internal I, feminine-masculine) ANOVA and with participants’ sex as moderator, where we expected a significant main effect of language form. When comparing the internal-I and feminine-masculine forms with the generic-masculine form, this should yield a higher number of women mentioned in the combined alternative forms. Participants’ sex is not of primary interest, as it showed only a small main effect and did not interact with the form factor in the original study.^6^ Moreover, we performed an additional multilevel analysis to take differences in the number of women between the celebrity categories within participants into account. Hence, we nested measures per celebrity category (level 1) in participants (level 2) and compared these results to the ANOVA results. The multilevel analysis was the focal analysis for Hypothesis 1.

In *Hypothesis 2*, we extended the original study (Stahlberg et al. 2001) in several ways: We added two additional celebrity categories to the original four to obtain more precision in the dependent variable (DV). Also, the language form factor contained five instead of three conditions (generic masculine, neutralized control, internal I, feminine-masculine, and gender star). Participant sex was treated as a covariate. We conducted a multilevel analysis with celebrity categories nested in participants and compared the generic masculine form and the neutralized control form with the gender-inclusive alternatives. We hypothesized that the gender-inclusive alternatives would yield a higher number of women mentioned than the generic-masculine and the neutralized control forms. This hypothesis was based on the findings of Pre-Study 1 and 2 that the generic masculine and neutralized control form yielded similar estimates.

In *Hypothesis 3*, which was based on Pre-Study 2, we predicted that a higher perceived base rate would be associated with a higher number of women mentioned when controlling for the form effect and participants’ sex. We again conducted a multilevel model and expected a language form effect (i.e., that more women should be mentioned when the gender-inclusive forms are used) from Hypothesis 2 to remain present.

Before we conducted any of the confirmatory multilevel analyses for Hypotheses 1 to 3, we tested if there was variance that could not be explained by the variables and nested structure of the multilevel models (i.e., residual heterogeneity). This would indicate that there might be variance across labs that needs to be accounted for, requiring us to add the labs as a third level to the multilevel structure.

Furthermore, in exploratory analyses, we tested the moderation of the effects of language form by several variables, including political orientation, attitudes toward gender-inclusive language, social-dominance orientation, and preference for socio-economic equality.

## Methods, Procedures, and Scales

### Power and Sample Size Justification

Our effect of interest in Hypothesis 1 is the contrast of the generic masculine versus its two original alternatives (internal-I and feminine-masculine form). As this is the relevant effect for the close replication of Stahlberg et al. (2001), we decided to base our power and sample size planning for the multi-lab study on this contrast. Importantly, this effect is based on approximately 60%^7^ of the total expected sample size, as the two additional groups (i.e., the gender-star form and neutralized control group) are not considered for Hypothesis 1. In this section, we describe how small the effect of the ANOVA contrast could be to be detectable with sufficient statistical power, while still being of practical relevance in our view. Thus, not only did we test whether people think of fewer female exemplars when exposed to the generic masculine compared to its two original alternatives, but we also employed equivalence testing to see if the difference is smaller than our smallest effect size of interest (SESOI; Lakens et al. 2018). The remaining multilevel models for Hypotheses 1 to 3 were then calculated using conventional null hypothesis significance testing criteria.

In the original study (Stahlberg et al. 2001, Experiment 2), a medium effect size was found for the relevant contrast, *d* = 0.59, with a large 95%CI of [0.14, 1.04], indicating low precision due to a small sample size of *N* = 90. Nonetheless, we assumed that our SESOI could be situated in the lower section of the original 95%CI. One method to determine a potential SESOI objectively is the small-telescope approach (Simonsohn 2015). This approach assumes that if one has a small chance of spotting an existing effect in an underpowered study (step 1), one should have a large chance of spotting the same effect in a sufficiently powered study (step 2). Hence, in step 1, we used information about the original study’s sample size (in our case: *n*_generic masculine_ = 30 and *n*_internal I and feminine-masculine_ = 60, see Stahlberg et al. 2001) to calculate which effect size could have been found with only 33% power (a threshold where studies are considered severely underpowered, see Simonsohn et al. 2014) and an α-error rate of 5%. In step 2, the resulting effect size from this sensitivity analysis (i.e., *d* = 0.34 as calculated in *JPower*, Morey 2020) would then be used for a sample-size calculation with sufficient power. A two-tailed *t*-test with *d* = 0.34, group-ratio = 0.5, power = 90%, and α-error rate = 5% yielded *n*_generic masculine_ = 112 and *n*_internal I and feminine-masculine_ = 224.

Although we consider *d* = 0.34 a realistic effect size given that our Pre-Study 2 has replicated the original study with a large effect (*d* = 0.78, see above), there remains the risk that the replication overestimated the true effect. Moreover, smaller, but potentially relevant effects could be involuntarily dismissed if assuming an effect size that is too large. We, therefore asked all participating labs for the total number of participants that would be feasible for them to recruit (see Table S1 in the Supplementary Document 1; https://osf.io/76un5/). Based on this, the overall number of participants that we could achieve is *N* = 3,150. When accounting for a participant exclusion rate similar to the pre-studies (~33%, yielding *N* = 2,100)^8^ and only considering the three groups for Hypothesis 1, we would obtain *N* = 1,260 (*n*_generic masculine_ = 420, *n*_internal I and female-male_ = 840) for the relevant contrast. Sensitivity analysis for a one-tailed *t*-test with these sample sizes, power = 90%, and false-positive rate = 5% indicated that we would find effects as small as *d* = 0.18 (Morey 2020; see Figure S1, https://osf.io/76un5/). This effect size would be smaller than the *d* = 0.34 from the small-telescope approach but still potentially practically relevant and it would still fall in the confidence interval of the original effect. Hence, *d* = 0.18 will constitute our SESOI for Hypothesis 1 and the minimal effect we are interested in (Lakens et al. 2018). The equivalence test to analyze whether our effect is statistically equivalent to a range from *d* = –0.18 to *d* = +0.18 would also be adequately powered (88%) for the sample size of *N* = 1,260 (Lakens 2018).

### Measures and Procedure

All materials can be found online (https://osf.io/f7ycs/). Participants were invited to participate in an eight-minute survey (LimeSurvey version 3, LimeSurvey GmbH 2021) on ‘the consumption of media and knowledge about celebrities.’ After giving their informed consent, they indicated whether they used a computer/laptop, a tablet, or a smartphone and whether their mother tongue was German. They were excluded from further participation if their mother tongue was not German. Similar to the original study (Stahlberg et al. 2001), the remaining participants filled out three distractor questions regarding media usage. These questions’ purpose was to strengthen the participants’ impression that this study was mainly about media consumption, but these responses were not investigated further.

Afterward, participants were asked to list famous people they know from the media. Specifically, they had to name three singers, athletes, politicians, TV hosts, authors, and actors/actresses. The latter two—authors and actors/actresses—were presented on a separate page to enable a close replication of Stahlberg et al. (2001), which is the focus of Hypothesis 1. Participants were randomly assigned to one of five forms of how the celebrities are presented (i.e., generic masculine, control, internal I, feminine-masculine, and gender star), which is the main independent variable. Our main dependent variable was the overall number of women mentioned (Hypothesis 1 [ANOVA]: 0–12) and the number of women mentioned per category (Hypothesis 1, 2 and 3 [multilevel models]: 0–3). Due to our experiences with incomplete data on the DV stemming from the pre-studies (i.e., participants did not always name at least two celebrities per category), we encouraged participants ‘to try to fill out all categories, as this is crucial for this study.’

Afterward, participants estimated the perceived base rate—i.e., to what degree men and women are present in the media—for each of the six celebrity categories on a scale from 1 (‘Men are much more present than women’) to 6 (‘Men and women are equally present’) to 11 (‘Women are much more present than men’).

On the next page, we presented participants with nine items measuring their attitudes toward gender-inclusive language (Sczesny et al. 2015). Responses were made on a Likert-type scale from 1 ‘applies not at all’ to 7 ‘applies very much’ (example item: ‘Using gender-fair language is important for me.’). As reliability evidence, we calculated the total Cronbach’s α and McDonald’s total ω, which were large in Pre-Study 2 (α = .94, ω = .94), after testing whether a unidimensional structure of the items fits the data (which was true for Pre-Study 2, χ²(27) = 194.54, *p* < .001), following the procedure proposed by Flora (2020). We used the scale mean in all relevant analyses.

Afterward, we showed participants the names of the celebrities they had listed before and asked them to count the number of females per category. This approach served two purposes: First, it provided a plausibility check as participants with implausible responses (i.e., above 3 or below 0) could be excluded. Second, we used those scores to ask them why they had mentioned only men if they typed ‘0’ in any category, or only women if they typed ‘3’ in any category. We also gave them the opportunity to explain additional reasons in a text field. We collected these data for descriptive purposes, and to see whether participants deliberately mentioned men and women due to the forms used in the manipulation (see also chapter ‘Internal-I confusion’: https://osf.io/ed5mv/).

On the next page, we collected information on the moderators and control variables (see Hypotheses section) participants’ sex, political orientation (11-point scale, from 1 ‘very left’ to 11 ‘very right’), social-dominance orientation (3 items, e.g., ‘Every society needs groups that are “on the top” and groups that are “at the bottom”.’ 5-point scale, from 1 ‘strongly disagree’ to 5 ‘strongly agree,’ see Aichholzer 2019), and preference for socio-economic equality (part of the Basic Social Justice Orientation scale, 3 items, e.g., ‘It is fair when all people have equal living conditions.,’ 5-point scale, from 1 ‘strongly disagree’ to 5 ‘strongly agree,’ see Hülle et al. 2017). We also collected additional information about the participants’ main residence during their childhood (village, small town, medium town, city/metropole), education, and nationality (all for descriptive/exploratory purposes). Finally, we asked to what degree they got distracted during the survey (1 ‘all the time,’ 2 ‘quite a lot,’ 3 ‘a little bit,’ 4 ‘not at all’) and whether they had participated in a similar study before (e.g., Pre-Study 1).

We excluded participants who did not pass the attention check (i.e., they report >3 or <0 women among their listed celebrities), who reported they got distracted ‘quite a lot’ or ‘all the time’ while filling out the survey, and who had taken less than four minutes to complete the study (half the time we expected). For testing Hypothesis 1 (as in Stahlberg et al. 2001), we excluded participants from the confirmatory analysis, if they named less than three persons per category. For testing Hypothesis 2 and 3 we excluded participants if they named less than two persons (Hypothesis 2 and 3; as in Pre-Study 2) per category. For testing Hypothesis 3 we excluded participants if they did not respond to the perceived base rate variables.

On the second-to-last page, we funnel-debriefed the participants with three questions, where the second and third questions dynamically appeared after the previous one was filled out. We asked participants 1) if they noticed something while listing the celebrities, 2) if they noticed something about the instructions for the celebrity-listing task, and 3) if (or how) they think the instructions for this task influenced their answering behavior. This funnel debriefing served as additional information but was not used as an exclusion criterion.

### Monitoring of the Data Collection

The data collection was planned to take place in twelve labs in parallel. All labs received access to identical versions of the online questionnaire. As we, the coordinating team consisting of the first and second authors, had full access to the data collection, we tried to ensure that data collection ended for each lab when 100% to 110% of the anticipated number of participants reached the end of the survey. Information on labs, including their location, the population they drew from (students in most cases), and participation incentives (if any) are provided in Table S1 (see https://osf.io/76un5/).

### Coding

After data collection, two independent raters^9^ coded all named celebrities based on four criteria (whether the text field was filled out; whether the person was a woman; whether the person was a man; or whether the response could not clearly be classified as man or woman). Moreover, the raters provided a reasoning for unusual cases in a separate column. Raters were not aware of the form condition (for detailed information, see https://osf.io/jk9mb/).

We evaluated the agreement among raters in percent. We expected nearly perfect rater agreement as our measure is relatively objective (counting the women among celebrities, coded 0 = ‘no,’ 1 = ‘yes’) which we indeed obtained (all κ ≥ .96). Disagreement in the remaining cases was resolved in discussions.

## Analyses

### Confirmatory Analysis

We tested Hypothesis 1, which is a close replication of Stahlberg and colleagues (2001, Experiment 2) by conducting a three-by-two ANOVA. We utilized the independent variables (IV) language form (generic masculine, internal I, and feminine-masculine) and participants’ sex (–0.5 = male, 0.5 = female) and applied Helmert-coded contrasts to the forms (see Table S2; for information on participants’ sex and the contrast schemes, see https://osf.io/76un5/). The number of women across the four celebrity categories was summed up (0 to 12), which constituted our DV. Using R version 4.3 (R Core Team, 2023), an ANOVA was performed for each participating lab. We extracted the contrast coefficient for the effect of interest (i.e., generic-masculine vs. internal-I and feminine-masculine form), the Cohen’s *d*s, as well as the means, standard deviations, and group sample sizes for each lab. Then, we calculated the meta-analytic summary effect for all extracted contrasts (based on the restricted maximum likelihood estimator in ‘metafor,’ version 3.0–1, Viechtbauer 2010, 2021) and tested it with regard to the SESOI of *d* = 0.18 (see power analysis above) using its 90%CI (which corresponds to a one-sided test). We only rejected the null hypothesis that people who are exposed to the generic masculine (compared to its alternatives) list the same number of women if the relevant difference was statistically different from 0. We rejected the hypothesis that an effect is statistically equivalent to zero, if it was not within the equivalence bounds of Δ*d* = ± 0.18. We used forest plots to depict the differences across labs. In line with the original study, we compared the marginal means of the three groups individually. We Bonferroni-corrected the α-error rate by the number of comparisons (3 means ≘ 3 comparisons), that is, α-error rate = .05/3 = .017. In this analysis, we used two-tailed tests against zero (i.e., we did not apply the SESOI). For the additional multilevel model, we applied the same procedure that we describe in the next paragraph but for the three original language form groups.

For Hypothesis 2, we conducted a multilevel analysis with a random-effects model (random intercepts and random slopes) as implemented in ‘lme4’ (version 1.1.-23, Bates et al. 2020). The DV was again the number of women mentioned but—in this case—in each of the five categories. We nested the measures for each category (level 1) in participants (level 2), resulting in our DV ranging from 0 to 3. As IVs, we used the language forms (level 2 predictor) and participants’ sex (level 2 predictor). Our contrast of interest (see Table S2, https://osf.io/76un5/)^10^ was the generic-masculine form and the neutralized control form versus the three alternatives (internal-I, feminine-masculine, and gender-star), where we expected that more women would be mentioned when the gender-inclusive alternative forms were used. The multilevel analysis was performed assuming Poisson-distributed data of the DV. This is more suitable for count data than assuming Gaussian-distributed data as it accounts for skew toward low numbers (Bates et al. 2015; Harris et al. 2014), which represented the distribution of our DV. Consistent with Hypothesis 1, we also checked for a potential interaction between sex and the language form, but our effect of interest remained the language form contrast described above. The relevant effects are shown in incident rate metrics (in line with the count data). For additional analyses, we extracted descriptive statistics for each condition per lab and across labs (e.g., for comparing means per form group; 5 means ≘ 10 comparisons).

For Hypothesis 3, the analysis plan followed the same procedure as for Hypothesis 2, but the perceived base rate (ranging from 1 = ‘Men are much more present than women’ to 11 = ‘Women are much more present than men’) was added as a level 1 predictor. Again, its interaction with the language form conditions variable was tested, but we were primarily interested in the main effects of both variables.

For all multilevel models, we applied conventional null hypothesis significance criteria (α-error rate of 5%). Although we assumed low (and non-significant) heterogeneity across studies, which was likely as all labs utilized the same online set-up (Olsson-Collentine et al. 2020), we tested for residual heterogeneity, as indicated in the hypothesis section. Residual heterogeneity was assessed based on the 95%CI of τ², where significant residual heterogeneity is present when this confidence interval does not cross 0. In this case, we planned to perform all multilevel analyses using labs as the third level (following participants and measures per category). We planned to further separate and examine samples from labs that may be responsible for residual heterogeneity.

### Exploratory Analysis

In several exploratory analyses, we wanted to expand on the extended replication model from Hypothesis 2 and 3. We examined whether political orientation (including social-dominance orientation and socio-economic equality preference) and attitudes toward gender-inclusive language showed main or moderation effects in generalized multilevel models^11^ predicting our main outcome. As for Pre-Study 2, we included the relevant contrast (generic masculine and neutralized control form vs. alternatives) together with these predictors and we also added interaction terms with the language forms. Further, we investigated the interplay with other covariates, such as sex and the perceived base rate. These exploratory analyses were performed with a split-half validation approach: We randomly split our final total sample, using the first half to identify relevant effects (based on an α error threshold of .05) and checking in the second half if these effects replicate.^12^

### Data Collection and Deviations from the Plan

#### Participants

We collected data between November 2021 and June 2022. Half of the labs could not reach their anticipated sample sizes (see Table 2). Importantly, sampling for the large Amazon Mechanical Turk sample by Metzler only reached 5% (n = 27/500). Hence, we reached out to the Leibniz Institute for Psychology (ZPID; https://leibniz-psychology.org/en/), which kindly supported the project by recruiting two large and diverse samples in Germany and Austria of *n* = 500 each via an external panel provider. A total of 3,816 people eventually participated in our study. In line with expectations, we had to exclude *N* = 837 of them for not meeting our preregistered data-quality-related criteria, leaving *N* = 2,979. For H2 and H3, we had to exclude 255 more because they did not give at least two responses in each celebrity category, leaving the sample for these analyses at *N* = 2,724. This would have left *N* = 1,494 for H1 (i.e., participants assigned to the groups generic masculine, internal-I, and feminine-masculine, who had provided responses in the relevant categories of singers, athletes, politicians, and TV hosts). Additionally, we excluded the data from our failed MTurk recruitment effort (27 valid responses, 15 of them relevant for H1). Our final samples of 1,479 people for testing H1 and 2,697 people for the remaining analyses were still slightly larger than anticipated, which is why we consider the study well-powered for our focal effects.

**Table 2:** Characteristics of the samples used to test the specific hypotheses.


LAB	n	GENDER			AGE	HIGHEST LEVEL OF EDUCATION					CHILDHOOD RESIDENCE				NATIONALITY				
				
		MEN	WOMEN	DIV.	M (SD); MIN–MAX	NONE	P/S SCHOOL	EXT. S SCHOOL	H/T SCHOOL	UNI.	VILLAGE	CITY	BIG CITY	METROP.	AT	DE	CH	OTHER	NA

**H1**																			

Bauch*	62	11	51	0	28.65 (10.08); 18–63	0	0	5	40	17	38	20	3	1	0	61	0	1	0

Beitner*	83	18	64	1	31.13 (9.67); 18–60	0	0	1	27	55	30	32	21	0	1	82	0	0	0

Brohmer & Hofer	125	70	55	0	35.96 (12.20); 20–71	0	0	1	22	102	69	29	24	3	118	4	0	3	0

Giuliani*	68	16	52	0	30.68 (11.56); 18–74	0	2	0	26	40	27	26	10	5	5	27	35	1	0

Gruber	121	31	90	0	25.69 (11.62); 18–73	0	1	1	87	32	65	35	14	7	57	60	0	4	0

Jauk	189	66	122	1	29.06 (13.85); 18–76	0	0	5	101	83	67	56	56	10	1	188	0	0	0

Malkoc*	56	28	28	0	30.70 (12.10); 18–65	0	0	0	23	33	35	14	5	2	50	3	0	1	2

Muees*	86	19	66	1	28.14 (8.83); 18–65	0	0	1	36	49	31	21	10	24	70	12	1	2	1

Salwender & Berkessel	217	42	173	2	25.37 (6.15); 18–63	0	0	2	107	108	101	62	40	14	2	210	0	2	3

Wehrt & Otto	124	31	93	0	27.90 (10.56); 18–64	0	0	2	76	46	60	49	10	5	1	122	0	1	0

ZPID-AT**	162	68	93	1	48.07 (14.28); 19–79	0	7	25	87	43	74	36	25	27	151	11	0	0	0

ZPID-DE**	186	73	112	1	53.56 (17.00); 18–87	0	12	52	68	54	56	76	40	14	1	184	0	1	0

Total	1479	473	999	7	34.08 (15.62); 18–87	0	22	95	700	662	653	456	258	112	457	964	36	16	6

**H2 & H3**																			

Bauch*	117	16	101	0	28.50 (9.74); 18–63	0	0	10	85	22	68	43	4	2	0	116	0	1	0

Beitner*	136	30	104	2	30.00 (8.52); 18–60	0	0	1	40	95	41	52	40	3	1	134	0	1	0

Brohmer & Hofer	224	121	102	1	37.32 (12.95); 20–100	0	0	4	38	182	133	50	37	4	209	11	0	3	1

Giuliani*	110	29	81	0	31.06 (11.68); 18–74	0	2	0	45	63	42	40	16	12	9	42	55	2	2

Gruber	227	56	171	0	25.08 (9.94); 18–73	0	2	1	167	57	117	64	33	13	99	123	0	5	0

Jauk	345	111	232	2	29.05 (14.19); 18–76	1	1	7	200	136	131	105	98	11	1	344	0	0	0

Malkoc*	111	54	57	0	31.05 (12.26); 18–71	0	0	0	44	67	65	32	10	4	99	8	0	2	2

Muees*	162	37	124	1	28.42 (9.41); 18–68	0	0	1	65	96	66	45	14	37	131	23	1	5	2

Salwender & Berkessel	375	79	293	3	25.90 (7.43); 18–79	0	0	5	190	180	166	120	67	22	2	367	0	2	4

Wehrt & Otto	232	52	177	3	27.85 (10.63); 18–68	0	0	3	146	83	103	92	27	10	1	228	0	2	1

ZPID-AT**	324	129	193	2	48.13 (14.39); 19–79	0	17	44	182	81	145	72	49	58	307	17	0	0	0

ZPID-DE**	334	139	192	3	53.89 (16.61); 18–87	1	26	94	115	98	90	138	80	26	2	330	0	2	0

Total	2697	853	1827	17	34.38 (15.79); 18–100	2	48	170	1317	1160	1167	853	475	202	861	1743	56	25	12


*Note*: Div. = Diverse; P/S school = primary or secondary school; Ext. S school = extended secondary school (‘Real-’ or ‘Mittelschule’); H/T school = high school diploma or trade school; Univ. = university degree; Metrop. = metropolis; AT = Austria; DE = Germany; CH = Switzerland; Other = other nationality (often double citizenship with either German or Austrian included); NA = response not provided. * Labs that could not reach the anticipated samples of n = 200 to 250; ** Additional samples to achieve the anticipated sample size.

Table 2 shows the final number of participants per lab together with some of their demographic data, separated for the sample used for testing H1 and the one used for testing H2 and H3. Our total sample consisted of more women than men and a small number of people selecting ‘other’ as gender. With a mean age of about 34 years, our total sample is older than a typical university student sample, with the average age of two additional panel samples being somewhat higher than the others. In line with our ethics agreement, our youngest participant was 18 years old. Our oldest participant reported an age of 100. In our sample the most common highest level of education was high/trade school, followed closely by a university degree. Most of our participants had grown up in towns or cities. Regarding nationality, we had more German than Austrian or Swiss participants. A small minority of people indicated ‘other’ as nationality, often specifying a double citizenship that included either German or Austrian. A few participants did not provide information regarding their nationality.

## Results

After we applied our preregistered data exclusion criteria, we double-checked the analysis code and statistical models with our team members. Despite the careful Stage 1 review, we noticed some shortcomings in our preregistered analysis plans and code. We address them and any changes we made to our plans in the sections below. We provide additional information on our analyses in the Results Appendices on descriptive statistics, reliabilities, and additional analyses (RA1), preregistered main analyses (RA2), and preregistered exploratory analyses (RA3) the OSF (https://osf.io/sqcrm/).

### Confirmatory Analysis

**Testing the effect of generic masculine vs. internal-I and feminine-masculine forms**. Our first test of Hypothesis 1 was analogous to the approach of the original authors (Stahlberg et al. 2001). The 2 (sex) × 3 (language forms) ANOVA resulted in a significant main effect of language form (*F*(2, 1473) = 190.72, *p* < .001, η_p_² = .206), a significant main effect of sex (*F*(1, 1473) = 93.57, *p* < .001, η_p_² = .06), and a non-significant interaction (*F*(2, 1473) = 2.11, *p* = .121, η_p_² = .002). Women named more female exemplars than men (*t*(1473) = 9.67, *p* < .001, *d* = 0.54). The contrast of interest (generic-masculine vs. internal-I and feminine-masculine form) also reached significance (*t*(1473) = 15.4, *p* < .001 *d* = 0.84). Additionally, its 90% confidence interval (90% CI*_d_* [0.75, 0.93]) fell outside of our equivalence bounds of Δ*d* = ± 0.18, leading us to reject the hypothesis that the effect of the generic masculine versus alternatives is equivalent to zero. Taken together, this can be viewed as evidence that people on average listed more women when gender-inclusive alternatives were used compared to the generic masculine. Therefore, we replicated the effect of Stahlberg et al. (2001).

As in the original study, we also compared the marginal means (*MM*) of the three groups individually. Both the internal-I (*MM* = 5.52, *SE* = 0.11) and the feminine-masculine forms (*MM* = 3.6, *SE* = 0.1) evoked more female exemplars than the generic masculine (*MM* = 2.47, *SE* = 0.1; internal-I vs. generic masculine: *t*(1475) = 21.09, *p* < .001, *d* = 1.37, 95% *CI* [1.23, 1.5]; feminine-masculine vs. generic masculine: *t*(1475) = 8.15, *p* < .001, *d* = 0.51, 95% *CI* [0.38, 0.63]). Additionally, the internal-I also yielded more female exemplars than the feminine-masculine form (*t*(1475) = 13.29, *p* < .001, *d* = 0.86, 95% *CI* [0.73, 0.99]).

**Figure 2:**
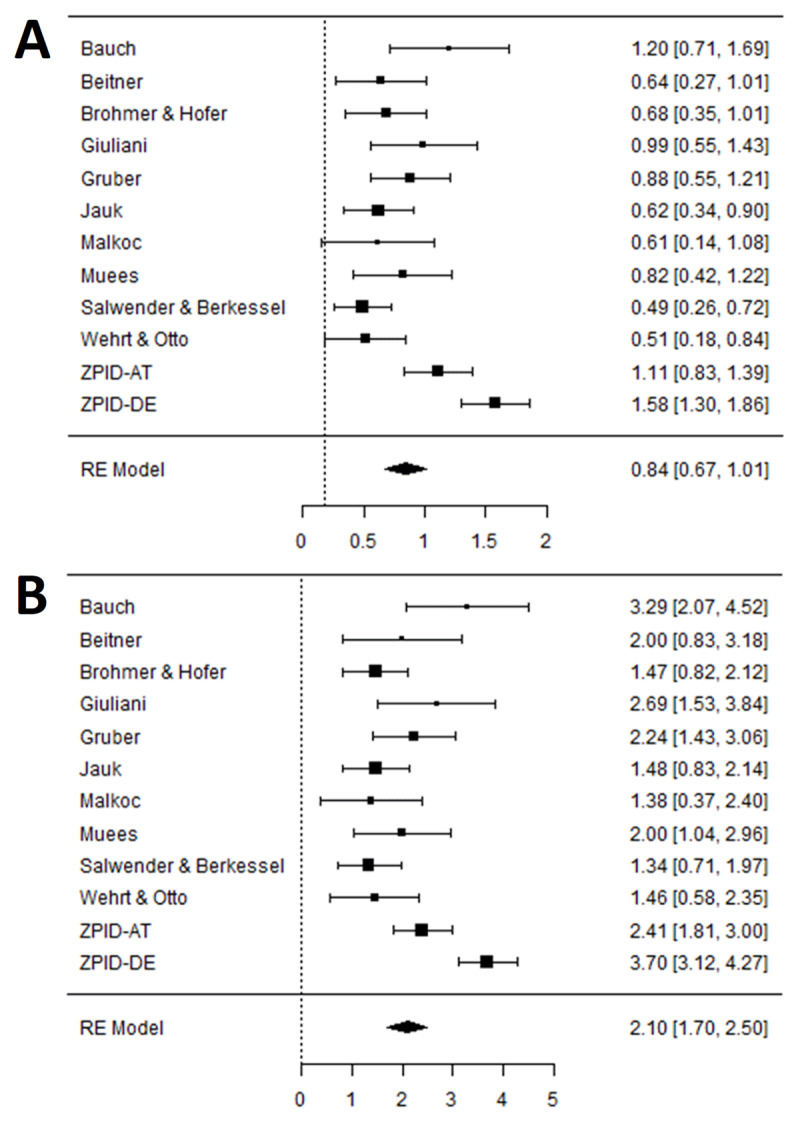
Forest Plots of the Main Contrast of Interest in Hypothesis 1 (Generic-Masculine vs. Internal-I and Feminine-Masculine Form). *Note*: *N* = 1479. Panel A shows the contrast expressed in the Cohen’s *d* metric. The vertical line represents our smallest effect size of interest (*d* = 0.18). Panel B shows the mean difference in the original metric (number of women mentioned; 0–12). The vertical line represents an effect of 0. Squares represent effects per lab with error bars being 90% confidence intervals (for one-sided testing). Diamonds are meta-analytic random effects.

Next, we ran the analyses testing Hypothesis 1 for each lab separately, extracted the results for the contrast of interest, and summarized them meta-analytically. Figure 2 shows meta-analytical findings as standardized (Panel A) and unstandardized mean differences (Panel B). The meta-analytic effect across all labs was again outside of our equivalence bounds, *d* = 0.84, 90% CI [0.67, 1.01]. Additionally, all point estimates of the contrast coefficients surpassed the threshold, although the confidence intervals of two labs crossed it.

Finally, we also tested Hypothesis 1 in a multilevel model to allow for a better representation of the structure of the data (participants nested in labs and categories nested in participants). Even though there was only little variance at the level of labs (both τ and *ICC* < .01), we included random intercepts for both levels to adequately model our data structure. We further wanted to include random slopes^13^ for the contrast-coded conditions as the effects of gender-inclusive forms might vary across labs. However, the model with random slopes had fit issues, evidence for benefits of including the slopes was mixed (random intercept model: *AIC* = 14690, *BIC* = 14730; random intercept and slope model: *AIC* = 14674, *BIC* = 14748; Likelihood-Ratio-Test: χ² (5) = 25.4, *p* < .001), and the inclusion of random slopes affected our main results only negligibly. Results of the random-intercept model were in line with the analyses reported above: Participants receiving prompts with gender-inclusive language named more female exemplars than those prompted with the generic masculine (*IRR*^14^ = 1.75, 95% CI [1.64, 1.87], *p* < .001). Among the gender-inclusive alternatives, the feminine-masculine form was associated with fewer women mentioned than the internal-I (*IRR* = 0.67, 95% CI [0.62, 0.71], *p* < .001). There was also an effect of participant sex: Women provided more female exemplars than men (*IRR* = 1.38, 95% CI [1.29, 1.48], *p* < .001). Detailed results including information on the two interactions with sex (which were non-significant for the contrast between the generic masculine and gender-inclusive alternatives but significant for the contrast between the two alternatives) can be found in Section 1.1.2.2 of RA2 (https://osf.io/ds3ag).

**Testing the effect of the generic masculine and control form versus gender-inclusive forms**. Next, we performed a multilevel analysis to test Hypothesis 2, examining whether the two alternative forms from H1 (internal-I and feminine-masculine) *and* the gender-star would prompt participants to come up with more female exemplars than the generic-masculine form *and* the neutralized control form. In all tested multilevel models, there was little to no variation on the lab level, 0.00 < τ < 0.01 (in the preregistered metric τ² ≤ 0.0001), .00 < *ICC* < .01, and some variation on the participant level, 0.09 < τ < 0.14, .16 < *ICC* < .18, which is in line with other multi-lab projects using identical materials (Linden & Hönekopp 2021; Olsson-Collentine et al. 2020). Still, we decided to keep both levels in our models to reflect our data structure adequately.

We conducted all analyses with two different contrast coding schemes: The first scheme (Hypothesis 2 REVISED A in Table S2) is a Helmert-scheme and close to what we preregistered. However, it does not allow for a direct test of our main contrast of interest (generic masculine & control vs. all gender-inclusive alternatives). For this reason, we introduced an alternative contrast coding (Hypothesis 2 REVISED B in Table S2, https://osf.io/76un5). As we see this contrast as the most direct test of our hypothesis, we focus on its results (for additional results on the other contrast coding, see Section 1.2.2 in RA2, https://osf.io/sqcrm/). We again tested a model including a random slope for this contrast, which showed better fit indices than a model with only random intercepts (random intercept model: *AIC* = 39862, *BIC* = 39900; random intercept and slope model: *AIC* = 39837, *BIC* = 39891; Likelihood-Ratio-Test: χ² (2) = 28.8, *p* < .001) but again had singular fit. We, therefore, again report the random-intercept-only model (for a plot of the random effects, see Section 1.2.1 in RA2, https://osf.io/sqcrm/).

The three gender-inclusive alternatives indeed yielded more female exemplars than the generic masculine and control condition, *IRR* = 1.50, 95%CI [1.44, 1.56], *p* < .001. Additionally, women mentioned more female exemplars than men did, *IRR* = 1.50, 95%CI [1.44, 1.57], *p* < .001. As preregistered, we ran another model including interactions with sex in addition to the main effects. There was a non-hypothesized interaction between participant sex and the relevant contrast, *IRR* = 0.91, 95%CI [0.83, 1.00], *p* = .043, indicating that the positive effect of the gender-inclusive forms was slightly *less* pronounced in women than in men. However, despite being statistically significant, this effect was small. For an overview of the number of named female exemplars per participant sex, category, and form, see Table 3.

**Table 3:** Mean number of women mentioned per sex, category, and group.


SEX	CATEGORY	*CONTROL* (*n* = 594)		*GENERIC M*. (*n* = 550)		*INTERNAL-I* (*n* = 473)		*FEM.-MASC.* (*n* = 551)		*GENDER S.* (*n* = 529)	
				
		*M*	*SD*	*M*	*SD*	*M*	*SD*	*M*	*SD*	*M*	*SD*

Male	Actor^+^	0.66	0.69	0.50	0.71	1.09	1.11	0.80	0.85	0.80	0.86

	Politician	0.80	0.71	0.68	0.62	1.21	0.94	0.82	0.64	0.85	0.77

	Singer	0.79	0.88	0.69	0.91	1.84	1.08	1.01	0.83	1.50	1.03

	Athlete	0.15	0.37	0.16	0.42	0.74	1.08	0.26	0.47	0.33	0.64

	TV host	0.54	0.64	0.57	0.76	1.05	1.04	0.69	0.76	0.72	0.82

	Writer^+^	0.52	0.66	0.40	0.63	0.96	0.98	0.64	0.73	0.67	0.76

Female	Actor^+^	1.02	0.82	0.84	0.86	1.49	0.97	1.18	0.82	1.18	0.92

	Politician	1.01	0.73	0.85	0.71	1.50	0.97	1.07	0.65	1.14	0.76

	Singer	1.22	0.91	0.98	1.02	2.31	0.87	1.60	0.91	1.90	0.95

	Athlete	0.45	0.66	0.30	0.60	1.08	1.11	0.53	0.72	0.62	0.89

	TV host	0.78	0.75	0.81	0.82	1.32	1.02	1.04	0.85	1.05	0.88

	Writer^+^	1.18	0.87	0.99	0.89	1.58	0.97	1.24	0.88	1.35	0.92


*Note*: *N* = 2,697. *SD* = standard deviation. ^+^ = category introduced for the extended replication. Possible range: 0–3.

**Figure 3:**
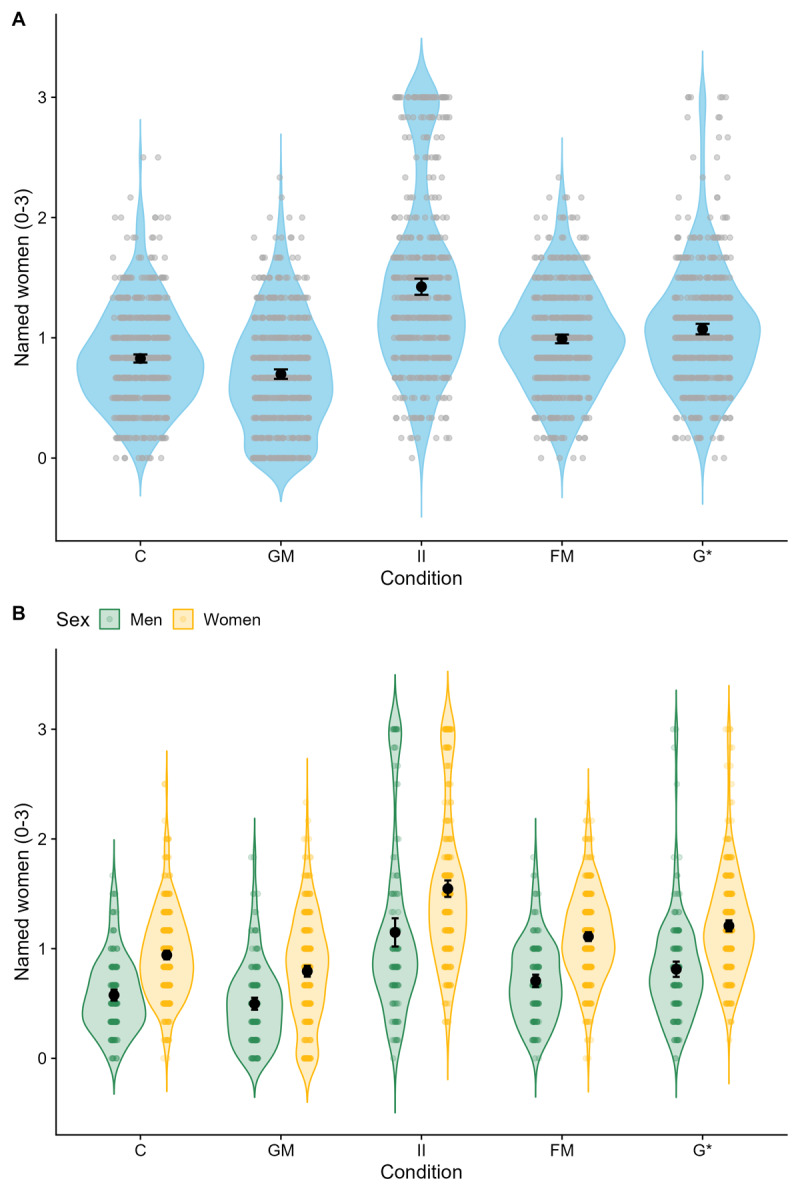
Violin plots showing the number of women named per condition **(A)** and per condition and sex **(B)**. *Note*: *N* = 2,697. Black dots indicate means and 95% confidence intervals. Colorful dots are participant-level data. C = control condition. GM = generic masculine. II = internal-I. FM = feminine-masculine. G* = gender star.

As preregistered, we also computed pairwise comparisons based on a model analogous to our main model for Hypothesis 2 but with participant sex and language form as factors (instead of planned contrasts). All pairwise comparisons were statistically significant after Bonferroni correction (all *p* ≤ .011; see Table 4). Participants prompted with the generic masculine mentioned fewer women than any other group (Figure 3; for additional descriptive statistics see Section 3.2 in RA1, https://osf.io/sqcrm/). Participants named significantly more women in the control group, followed by the female-male group, and which was in turn followed by the gender-star group. Participants in the internal-I condition named the most women.

**Table 4:** Ratios of differences in the number of named women between conditions (pairwise comparisons).


CONTRAST	RATIO	*SE*	*z*-RATIO	*p*

C vs. GM	1.19	0.04	5.50	<.001

C vs. II	0.59	0.02	–18.38	<.001

C vs. FM	0.84	0.02	–5.79	<.001

C vs. G*	0.77	0.02	–9.02	<.001

GM vs. II	0.49	0.02	–23.01	<.001

GM vs. FM	0.71	0.02	–11.04	<.001

GM vs. G*	0.64	0.02	–14.11	<.001

II vs. FM	1.44	0.04	12.66	<.001

II vs. G*	1.31	0.04	9.38	<.001

FM vs. G*	0.91	0.03	–3.27	.011


*Note*: *N* = 2697. C = control condition. GM = generic masculine. II = internal-I. FM = feminine-masculine. G* = gender star. *p*-values are Bonferroni-corrected (adjusted for 10 tests). Tests were performed on the log scale. Values above 1 indicate a higher number of women named in the left compared to the right condition (e.g, in the first row, more people were named in the C than in the GM condition).

**Figure 4:**
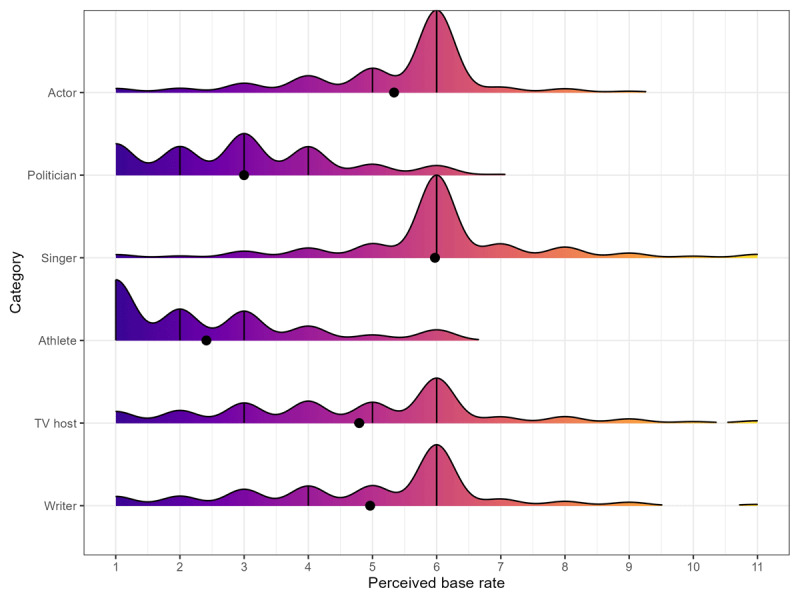
Ridgeline plots showing the perceived base rate per category. *Note*: *N* = 2,697. Black dots indicate means and 95% confidence intervals (not visible here, due to precise estimation). Black horizontal lines indicate quartiles. The scale ranged from 1 (‘Men are much more present than women’) to 11 (‘Women are much more present than men’).

**Testing perceived base rates**. To test Hypothesis 3, we performed a similar analysis as for Hypothesis 2 (REVISED B) but added participants’ perceived base rates for each celebrity category as covariate. On a scale ranging from 1 (‘Men are much more present than women’) to 11 (‘Women are much more present than men’), the average ratings of the perceived base rate fell between *M* = 2.41, *SD* = 1.58 (athlete) and *M* = 5.97, *SD* = 1.69 (singer; for an overview see Figure 4). Thus, although perceived base rates varied across conditions, participants, on average, thought that men were more present than women across celebrity categories. The exception was the category of singers, where the average response and its confidence intervals were close to 6 (‘Men and women are equally present’). In addition to random intercepts and a random slope for our contrast of interest at the lab level, we first also included a random slope for the perceived base rate at the participant level (as the association between perceived base rate and number of women named might differ between participants). However, this model had a singular fit, so we could not interpret it. The model with the random slope for our contrast of interest had superior fit to the model without random slopes (random intercept model: *AIC* = 38774, *BIC* = 38820; random intercept and slope model: *AIC* = 38751, *BIC* = 38812; Likelihood-Ratio-Test: χ² (2) = 27.3, *p* < .001) and is the basis of our interpretation. The effects of gender-inclusive forms, *IRR* = 1.47, 95%CI [1.36, 1.59], *p* < .001, and participant sex, *IRR* = 1.54, 95%CI [1.48, 1.62], *p* < .001, remained significant but the perceived base rate also showed a small effect, *IRR* = 1.13, 95%CI [1.12, 1.14], *p* < .001. Participants who indicated that more women were present in a given category also mentioned more women in the main task. Next, we added an interaction term between gender-inclusive forms and perceived base rate. Its effect was small but significant, *IRR* = 0.97, 95%CI [0.96, 0.99], *p* = .001, indicating that a higher perceived base rate of women was associated with a lower effect of gender-inclusive alternatives on the number of women mentioned.

### Preregistered Exploratory Analysis

As planned, we explored the following participant variables as potential predictors of the number of women mentioned and as moderators of the effect of language form on the number of women mentioned: Attitudes toward gender-inclusive language, political orientation, social-dominance orientation, and preference for socio-economic equality. We checked for reliability evidence before computing aggregate scores, following the procedure by Flora (2020). The attitudes toward gender-inclusive language scale fit a unidimensional structure according to most indices (robust fit statistics: *CFI* = .97, *TLI* = .96, *RMSEA* = .1, *SRMR* = .03) and the corresponding reliability indicator ω_u_ of .95 pointed toward high reliability. As the scales for social dominance orientation and equality preference encompassed three items only, unidimensionality could not be tested following this approach. We still computed Cronbach’s α and McDonald’s total ω as reliability evidence, which were in the range one would expect for three items (social dominance orientation: α = .61, ω_total_ = .62; preference for socio-economic equality: α = .69, ω_total_ = .71). We further computed descriptive statistics and intercorrelations of the participant variables (see Section 5 in RA1, https://osf.io/sqcrm/). The intercorrelations were moderate to high (*r*s between |.25| and |.50|) and in the directions one would anticipate for these constructs (e.g., more positive attitudes toward gender-inclusive language were associated with a more left political orientation, a higher preference for socio-economic equality, and a lower social-dominance orientation).

Analogously to our analyses for the effects of perceived base rate, we ran two multilevel models for each moderator (Model 1 includes the main effect of the moderator together with the main effects of participant sex and the deviation-coded contrast; Model 2 additionally includes the interaction terms between the moderator and the deviation-coded contrast). We included random intercepts but not slopes, due to theoretical considerations. Here, we only report results for the main effects of the moderators and their interactions with the deviation-coded contrast (REVISED B; for detailed results see RA3, https://osf.io/sqcrm/). Following our preregistration, we randomly split our sample into a training and validation dataset. To avoid oversampling from a given lab or condition, we sampled from each condition/lab separately.

More positive attitudes toward gender-inclusive language were associated with naming more women. This main effect held in both the training (*IRR* = 1.04, 95%CI [1.02, 1.05], *p* < .001) and the validation data set (*IRR* = 1.05, 95%CI [1.03, 1.06], *p* < .001). There was no interaction effect between attitudes toward gender-inclusive language and language form in either of the two data sets (training: *IRR* = 0.97, 95%CI [0.94, 1.01], *p* = .099; validation: *IRR* = 0.99, 95%CI [0.96, 1.03], *p* = .700).

For political orientation, main effects in both data sets suggested that participants leaning more toward the right named fewer women (training: *IRR* = 0.98, 95%CI [0.96, 0.99], *p* = .007; validation: *IRR* = 0.98, 95%CI [0.96, 0.99], *p* = .006). There were no significant interaction effects (training: *IRR* = 1.02, 95%CI [0.98, 1.05], *p* = .298; validation: *IRR* = 1.02, 95%CI [0.98, 1.05], *p* = .331).

Social-dominance orientation yielded inconclusive results for a main effect (training: *IRR* = 0.98, 95%CI [0.94, 1.02], *p* = .335; validation: *IRR* = 0.95, 95%CI [0.92, 0.99], *p* = .013). The interaction effect was consistently non-significant (training: *IRR* = 1.06, 95%CI [0.98, 1.15], *p* = .152; validation: *IRR* = 1.01, 95%CI [0.94, 1.08], *p* = .821).

Finally, the higher participants’ preference for socio-economic equality, the more women they mentioned in the training dataset, but this was not the case in the validation dataset (training: *IRR* = 1.05, 95%CI [1.02, 1.08], *p* = .002; validation: *IRR* = 1.02, 95%CI [0.99, 1.06], *p* = .111). There was no associated interaction with the main contrast (training: *IRR* = 1.05, 95%CI [0.98, 1.11], *p* = .170; validation: *IRR* = 0.99, 95%CI [0.93, 1.06], *p* = .794).

Taken together, all reported exploratory effects were smaller than the gender-inclusive form effects from the main findings of the confirmatory analyses.

### Additional Exploratory Analyses

In addition to our planned analyses, we explored cases in which participants only mentioned women or men, respectively, across all 18 possible responses. Out of the total valid sample of *n* = 2,697, *n* = 46 participants only named women. Forty of them were in the internal-I condition (8% of participants in this condition) and six were in the gender-star condition (1% of participants in this condition). We asked them for their reasons (selecting multiple was possible) for only naming women for any category where they had done so. The majority (37 to 42 per category) reported that they had understood the instruction as specifically asking for women. Some responded that they had just reported what had come to their mind spontaneously (2 to 4 per category) or that they just did not have any men in mind (2 to 3 per category). No person selected not having known any men in the respective category as the reason. Thus, a minority seemed to have interpreted two of our gender-inclusive alternatives as specifically asking for women.

We further assessed participants who did not name a single woman in any condition (*n* = 81 participants in total). While predominantly being in the generic masculine condition (*n* = 64, 12% of participants in this condition), they were also present in all other conditions (control: *n* = 10, internal-I: *n* = 1, feminine-masculine: *n* = 4, gender star: *n* = 2). Compared to those only naming women, the reasons these participants described were more diverse: While the majority (53 to 54 per category) reported that they had understood the instruction as specifically asking for men, a considerable number also responded that they had just reported what had come to their mind spontaneously (19 to 22 per category). Between five and seven per category responded that they had no women in mind. For the categories athlete (3), TV host (2), and writer (2) some participants also indicated not knowing any women as the reason. Thus, while some participants interpreted the generic masculine non-generically and, therefore, only named men, others might have struggled to come up with female celebrities in general.

To get an impression of the effect of wrongfully interpreting the generic masculine as only referring to men or the internal I as only referring to women, we re-ran the pairwise comparisons for Hypothesis 2 after excluding participants who only named men or women, respectively, and who responded that this was how they had understood the instruction (*n* = 127). In this analysis, the internal I remained the condition associated with the highest number of women named and all gender-inclusive alternatives were still associated with more female exemplars listed than either the control condition or the generic masculine (all *p* < .001). The only discrepancies to the results for the full sample were that differences in female exemplars listed in response to the generic masculine versus the control condition (*p* = .299) and feminine-masculine word pairs versus the gender star (*p* = .069) were no longer significant (for all details see Section 6 in RA1, https://osf.io/sqcrm/).

## Discussion

Despite the rise of gender-inclusive alternatives, the generic masculine form remains highly prevalent in German-speaking countries (e.g., Waldendorf 2024). Public debates about the use of gender-inclusive language often result in heated discussions (see von Blazekovic 2021; Hanfeld 2022; Schmoll 2022). These controversies stand in contrast to a body of research demonstrating that the language we use to describe people may affect how they are perceived (e.g., Sczesny et al. 2016, but also see IJzerman et al. 2015). In the present project, we aimed to replicate and extend a classic finding from over 20 years ago (Stahlberg et al. 2001). As in this original study, we found that when asking individuals to name celebrities such as singers or politicians, using gender-inclusive alternatives leads to a higher number of women being mentioned than using the generic-masculine form. Our replication effort spanned twelve labs in Austria, Germany, and Switzerland, and included two large and diverse samples from Germany and Austria.

### Close Replication of Stahlberg et al. (2001)

First, we found that when participants were prompted with the gender-inclusive alternatives (internal-I and female-male word pairs), they named more female singers, athletes, politicians, and TV hosts compared to the generic masculine, confirming our first hypothesis and aligning with the original findings. Compared to the original effect (*d* = 0.59, 95%CI [0.14, 1.04]), our meta-analytical effect was slightly larger (*d* = 0.84, 90%CI [0.67, 1.01]) although there was also some variability with effects ranging from *d* = 0.49 to *d* = 1.58 across labs (see Figure 2). Moreover, the positive effect of gender-inclusive alternatives also held in a multi-level model accommodating participants’ variability in naming women across labs and categories. Overall, the effect of language form was therefore replicated. We find this result notable, particularly because the original finding was obtained over 20 years ago. Some have argued that people have become more accustomed to gender-inclusive alternatives (e.g., Waldendorf 2024), potentially limiting the effectiveness of their use. The results of this multi-lab study, however, suggests that using gender-inclusive alternatives was still effective in bringing women to people’s mind.

### Extension of Stahlberg et al. (2001)

For the extension, we added two more conditions to the original design, enabling us to test our second hypothesis. The neutralized form served as a true control condition. It allowed us to determine whether the generic masculine leads to more men being named or whether gender-inclusive alternatives leads to more women being named. The gender star is a more recent alternative than the internal I and female-male-word pairs (see Waldendorf 2024) and aims to also include non-binary and gender-diverse people. We also added two categories of celebrities—writers and actors—to offer more possibilities to mention celebrities from the cultural landscape. When we compared the generic masculine and neutralized control form to the gender-inclusive alternatives in this extended design, the latter conditions resulted in more women being mentioned. This pattern clearly confirmed our second hypothesis.

In pairwise comparisons, all gender-inclusive alternatives led to more women mentioned, not only compared to the generic masculine but also to the neutralized form. This means that participants named more women when prompted in a form that explicitly referred to any gender that is not male than when the prompt did not include information about gender. Still, the generic masculine form resulted in somewhat fewer women being named than the neutralized control form. Taken together, gender-inclusive alternatives like the internal I, feminine-masculine word pairs, or the gender star seem to actively encourage people to come up with women, whereas the generic masculine appears to reduce the number of women mentioned, potentially by activating male mental representations. Moreover, our exploratory analyses suggested that some people may also actively understand the generic masculine as only referring to men.

When comparing the different gender-inclusive alternatives, feminine-masculine word pairs resulted in the lowest number of female exemplars, closely followed by the gender-star. The condition that was associated with the highest number of women named was the internal I. In this condition, approximately one-half of the responses contained names of women. This finding is also in line with another study that replicated Stahlberg et al. (2001) and was published while the present study was in progress (Keith et al. 2022). It is currently not completely clear why the internal I was more effective in increasing female responses than the other gender-inclusive alternatives. Based on our exploratory analyses, some people might mistakenly assume that the internal-I form means that they should only name women (i.e., they interpret it non-generically). One reason for this might be the morphological similarity of the internal I and feminine-only forms (e.g., the minimal difference between DoktorInnen and Doktorinnen). However, even when we excluded these participants from the analyses, the internal-I form remained associated with the highest number of women named.

In the final part of our preregistered extension, we investigated the role of the perceived base rate—the assumed proportion of men or women in the respective category. Importantly, the effect of gender-inclusive language remained present when controlling for participants’ estimates of the perceived base rate, speaking to the robustness of this effect. The perceived base rate itself also showed a small effect. Specifically, if participants assumed that a higher proportion of women was present in a celebrity category, they also tended to name more women in this category. Moreover, there was a small but significant moderation effect, indicating that a higher perceived base rate was associated with a less pronounced effect of language form. Thus, gender-inclusive alternatives were more effective in prompting more female exemplars when people thought that a lower proportion of women was present in a category. Notably, participants on average reported a higher perceived base rate of men across all conditions apart from singers, where the average response indicated that participants believed that men and women were about equally common. It would be interesting to see whether the positive effects of gender-inclusive alternatives on recalling women also holds in domains that people associate with a higher proportion of women than men.

### Individual Differences in the Tendency to Mention Women and Related Variables

In addition to the main analyses on the replicability of the effect of gender-inclusive language, we explored different variables (participant sex, attitudes toward gender-fair language, or political orientation including social-dominance orientation, and preference for socio-economic equality) that may be related to individual differences in the overall tendency to mention women when asked about celebrities. In line with the original study (Stahlberg et al. 2001), one significant variable was participant sex. That is, independently of the condition participants were in, women named more women than men did. Moreover, more positive attitudes toward gender-inclusive language and a more left-leaning political orientation were also associated with naming more women. All these effects were descriptively smaller than the effect of language form and did not affect its significance.

We further tested whether any of our individual difference variables moderated the effect of gender-inclusive alternatives. Only participant sex was a significant moderator: The positive effect of gender-inclusive languages compared to the generic masculine and control condition was less pronounced in women than in men. Importantly, the interaction only reached significance in one out of our multiple analyses and was also not present in the original study (Stahlberg et al. 2001), raising questions about the robustness of this effect.

Overall, the general absence of interaction effects in our study may indicate that gender-inclusive language is effective in bringing female exemplars to people’s minds irrespectively of their political orientation or whether they have positive attitudes toward such language (cf. Stahlberg & Sczesny 2001). However, one should keep in mind that we based our sample size planning on the main effect of language forms, as this was the focal effect in our study. Because interactions are far more power-intensive than main effects (e.g., Sommet et al. 2023), it is likely that a higher participant number would have been necessary to draw definite conclusions about the absence of interactions.

### Implications

Our study shows that the positive effect of using gender-inclusive language in prompting people to think of women replicates even after twenty years, a period during which society has become more accustomed to such language. This finding aligns with a substantial body of literature indicating that the generic masculine is not always perceived or understood generically (Braun et al. 2005; Keith et al. 2022; Vervecken et al. 2013).

Additionally, results from this confirmatory report—particularly the similarity of effects for the neutralized control form and the generic masculine—imply that people think of men rather than women when naming celebrities. The perceived base rate ratings, which did not favor women in any celebrity category, support this narrative. Taken together, these results suggest that male exemplars are often considered as the default in these categories, which might be due to stereotypes about what makes a successful public figure. In general, these stereotypes may come from a general androcentric bias (Bailey et al. 2019; Davis 2021), which is the tendency to associate human beings and their needs first and foremost with men. Moreover, the celebrity categories that we focused on here are also subject to gender inequality (see e.g., Bielby 2014). Gender-inclusive language (e.g., in media reports) could have positive effects on women’s representation in these fields and increase the visibility of highly successful women in the long run.

Our findings are highly relevant considering recent controversial debates (see von Blazekovic 2021; Hanfeld 2022; Schmoll 2022) and legislative developments in Austria and Germany, where conservative governments have restricted the use of gender-inclusive language in public institutions (e.g., in Bavaria or Lower Austria, see Bayerische Staatsregierung 2024; NOE 2023). Specifically, many of these regulations explicitly ban the use of the internal-I and non-binary inclusive language like the gender star, instead suggesting feminine-masculine word pairs (i.e., the least effective alternative form in our study) or neutral forms (which we found to be only slightly better than the generic masculine). In principle, the introduction of general guidelines for gender-inclusive language could facilitate its application and increase the visibility of women. However, our data suggest that the internal I and the gender star may be more effective in bringing women to people’s minds than the alternatives proposed in these recent regulations. The gender star also has the additional benefit of acknowledging non-binary or gender-diverse individuals (e.g., Pfadenhauer 2024), and thus, may enhance their representation in language.

### Strengths and Limitations

In our study, we confirmed the positive effects of gender-inclusive alternatives on the cognitive inclusion of women in a large-scale, preregistered replication effort across multiple Austrian, German, and Swiss labs. By including the original authors in our planning, we were able to conduct a close replication that only had negligible discrepancies to the original design. For our extended replication, we considered additional conditions and celebrity categories to solidify the conclusions we could draw from our results. Our main results held across the original and the extended design and across different analytical specifications.

Despite these strengths, some limitations need to be considered when interpreting our results. First, the largest discrepancy between our study and the original one (cf. Stahlberg et al. 2001) is that we collected data online instead of in a laboratory. On the one hand, our large data collection effort would likely have taken much longer in an in-person setting. On the other, we had no control over potential distractions or the use of unwanted helpers (e.g., a search engine) during our naming task. Nonetheless, our strict exclusion criteria to control for distraction and the fact that participants had nothing to gain from cheating (i.e., looking up celebrities), still speak for a high data quality.

Our second set of limitations refers to sampling. We had unanticipated sampling problems with Amazon Mechanical Turk and had to make modifications to our sampling strategy. However, the two additional samples we recruited via the ZPID still enabled us to test our hypothesis with adequate statistical power for our confirmatory hypotheses. Moreover, the two additional samples were rather diverse. Still, when considering our results, and particularly those on individual differences, readers should bear in mind that we obtained them in samples that were mostly homogeneous.

Our third set of limitations refers to the conclusions we can draw from our design. We cannot determine the exact cognitive processes that lead people to name fewer women when prompted in the generic-masculine form. For instance, we do not know whether the processes are automatic or the result of deliberate thinking (e.g., if gender-inclusive prompts lead people to not only think ‘I should name singers’ but also ‘My responses should also include women,’ potentially to comply with an external moral appeal, see e.g., Lipsitz 2018). Moreover, although we included random intercepts for celebrity categories, our findings may not necessarily generalize to the mental representation of men and women of other areas beyond celebrity categories. After all, when thinking about members of other occupations (like doctors, researchers, engineers, and so on), it is likely that personal relationships play a larger role in activating these representations than when thinking about prominent figures from the media. Finally, our study focused on the inclusion of women due gender-inclusive alternatives. However, future work is needed to investigate whether gender-inclusive alternatives also increase the mental inclusion of non-binary people. This may be particularly important given the current political efforts trying to restrict the use of alternatives that include genders outside the binary.

## Conclusions

Using gender-inclusive language can have positive effects on women being represented in readers’ minds compared to using the generic masculine. Moreover, using the generic masculine results in even fewer women being named than not mentioning gender at all. In other words, when someone wants to refer to men and women, using masculine forms is not a suitable way to achieve this. The beneficial effect of using gender-inclusive alternatives replicated even after 20 years and persisted after controlling for participants’ sex, perceived base rates of women in the respective celebrity category, and political orientation. The individual differences we investigated seem to play a negligible role in the effectiveness of gender-inclusive language in raising the availability of female exemplars. Although the evidence we reported here is restricted to this specific effect, our results consistently demonstrate that gender-inclusive language is effective in encouraging recipients to be more aware of female representatives of different celebrity categories. We suggest that official recommendations on the use of gender-inclusive language should be based on solid scientific evidence. Clearly, the findings we report in this study would certainly contribute to sound evidence-based policy making.

## Data Accessibility Statement

As part of this registered confirmatory report, all materials and data can be found online and are listed here:

– Original project folder: https://osf.io/fdrn6/ and https://doi.org/10.23668/psycharchives.6532
– In-principle accepted manuscript: https://osf.io/knpxt
– Materials: https://osf.io/ngjb2/
– Data and code: https://osf.io/sqcrm/ and https://doi.org/10.23668/psycharchives.8416
– Supplemental Materials 1: https://osf.io/76un5
– Supplemental Materials 2: https://osf.io/ed5mv
– Supplemental Materials 3: https://osf.io/ecpgx

## Footnotes

This exemplar retrieval process from memory is assumed to be rather spontaneous/automatic and less controlled when the concept of ‘man’ is activated in the associative network (Shiffrin & Schneider 1977; but see also Amodio 2019; Fiedler & Hütter 2014). Although the precise cognitive mechanisms for these retrieval processes (see Barsalou 2003) are not well specified in the context of gender-inclusive language use (Braun et al. 2005), the outcome—an underrepresentation of women—is evident and of primary interest.

In German ‘Binnen-I’; there is no current standard notation for this medial capitalized form, which is conceptually comparable to the so called ‘camel case’ in English language (Cambridge Dictionary 2021).

In German language, there are even more gender-inclusive alternatives, such as the gender gap (e.g., ‘Politiker_innen’), gender slash (‘Politiker/innen’), or the gender colon (e.g., ‘Politiker:innen’). Here, we will confine ourselves to the gender-star complementing the internal-I and feminine-masculine form.

Based on the reported p-value, *p* < .01 (conservatively rounded to .01), and information on the sample size, *N* = 90, we calculated this effect using the R package *compute.es* (version 0.2–5, Del Re, 2020) pes(p = .01, n.1 = 30, n.2 = 60, tail = “two”).

In line with the original study and to avoid confusion with our experimental manipulation (i.e., gender-inclusive forms) and other predictors (e.g., attitudes for gender-inclusive language), we refer to participants’ self-reported gender identity as ‘sex.’

Further, as in most non-representative studies, participant sex was not balanced in our study, which limits interpretations.

In our final analyses, the total sample for H1 was 54.8% of the size of the one for H2.

Importantly, in Pre-Study 2, we excluded smartphone users from participating and they accounted for approximately 20% of all participants. As we adjusted the survey settings in favor of smartphone users (see next section), we expected a much lower exclusion rate and a larger total sample. For instance, if the exclusion rate was 20% instead of 33%, we would get *N* = 1512 (*n* = 504 per group). For a one-tailed *t*-test with power = 90% and α-error rate = 5% we could find *d* = 0.16.

As rater-burden was relatively high, we had a total of seven raters, with two of them always doing the ratings for a given lab (i.e., coding between 522 and 10,800 individual responses).

Note that in the preregistration, we indicated that the contrast of interest is part of a forward-difference coding scheme. However, as this contrast would not adequately test Hypothesis 2, we reformulated the coding scheme. All changes can be found in Table S2.

In the preregistration, we wrote that we would test for these effects in ‘general linear models and multilevel models.’ Instead, we used generalized multilevel models for all of these analyses because they all used the same count data.

In the preregistration we stressed that ‘we will test Hypothesis 1 to 3 again by including participants that were previously excluded (see “Methods and Procedures”).’ However, our exclusion criteria encompassed multiple steps, differed across hypotheses, and—above all—were based on criteria that ensured high data quality. Rerunning all analyses would have meant including data of dubious quality (e.g., by inattentive participants) and would have further resulted in the need to make additional analytic decisions based on these results (e.g., which random effects to include). Thus, we refrained from running these analyses. The full dataset is available online for interested readers (https://doi.org/10.23668/psycharchives.6532).

In our preregistration we did not specify which random slopes we would model. For this reason, we included random slopes based on theoretical considerations (i.e., gender-inclusive alternatives might have different effects in different areas), while aiming to avoid overfitting and model convergence issues.

In our preregistration, we did not specify the type of unstandardized coefficient we would report. As we had Poisson-distributed data, we decided to report the incidence rate ratio (*IRR*), which can be interpreted similarly to the odds ratio (i.e., 1 corresponds to a null effect; see Imran et al. 2022). To aid our interpretation, a Cohen’s *d* of ± 0.18 (a small effect, which is in line with our equivalence threshold for H1) would be similar to an *IRR* = 0.72/1.38.
